# *Yersinia pestis* can infect the Pawlowsky glands of human body lice and be transmitted by louse bite

**DOI:** 10.1371/journal.pbio.3002625

**Published:** 2024-05-21

**Authors:** David M. Bland, Dan Long, Rebecca Rosenke, B. Joseph Hinnebusch

**Affiliations:** 1 Laboratory of Bacteriology, Rocky Mountain Laboratories, National Institute of Allergy and Infectious Diseases, NIH, Hamilton, Montana, United States of America; 2 Rocky Mountain Veterinary Branch, Rocky Mountain Laboratories, National Institute of Allergy and Infectious Diseases, NIH, Hamilton, Montana, United States of America; Institut Pasteur, FRANCE

## Abstract

*Yersinia pestis*, the causative agent of plague, is a highly lethal vector-borne pathogen responsible for killing large portions of Europe’s population during the Black Death of the Middle Ages. In the wild, *Y*. *pestis* cycles between fleas and rodents; occasionally spilling over into humans bitten by infectious fleas. For this reason, fleas and the rats harboring them have been considered the main epidemiological drivers of previous plague pandemics. Human ectoparasites, such as the body louse (*Pediculus humanus humanus*), have largely been discounted due to their reputation as inefficient vectors of plague bacilli. Using a membrane-feeder adapted strain of body lice, we show that the digestive tract of some body lice become chronically infected with *Y*. *pestis* at bacteremia as low as 1 × 10^5^ CFU/ml, and these lice routinely defecate *Y*. *pestis*. At higher bacteremia (≥1 × 10^7^ CFU/ml), a subset of the lice develop an infection within the Pawlowsky glands (PGs), a pair of putative accessory salivary glands in the louse head. Lice that developed PG infection transmitted *Y*. *pestis* more consistently than those with bacteria only in the digestive tract. These glands are thought to secrete lubricant onto the mouthparts, and we hypothesize that when infected, their secretions contaminate the mouthparts prior to feeding, resulting in bite-based transmission of *Y*. *pestis*. The body louse’s high level of susceptibility to infection by gram-negative bacteria and their potential to transmit plague bacilli by multiple mechanisms supports the hypothesis that they may have played a role in previous human plague pandemics and local outbreaks.

## Abstract 2

Yersinia pestis, the causative agent of plague, uses fleas and rodents as vectors for transmission. This study reports a new vector for Y. pestis, showing that it can infect the Pawlowsky glands of human body lice and be transmitted by louse bite.

## Introduction

Human body lice, *Pediculus humanus humanus*, are obligate blood-feeding parasites of their sole host, humans. The insects reside within the seams of human clothing where they lay their eggs, periodically returning to the skin to feed 5 to 6 times per day [1]. Present day infestation by body lice is typically associated with homelessness, poverty, war, and natural disaster; any situation that results in reduced hygiene and access to clean clothing and bedding. However, *Pediculus* are estimated to have become parasites of our hominid ancestors 5 to 6 million years ago and, for most of human history, parasitization was commonplace [2].

The current scientific consensus is that human body lice are vectors of 3 bacterial pathogens: *Bartonella quintana* (Trench Fever), *Borrelia recurrentis* (Louse-borne relapsing fever), and *Rickettsia prowazekii* (Epidemic Typhus) [3]. Transmission of these pathogens primarily occurs by bacteria-laden feces or fluids from crushed lice being rubbed or scratched into abrasions on the skin [4]. While body lice may harbor other opportunistic bacterial pathogens such as *Acinetobacter* spp. and *Serratia marcescens* [5], they have not been linked to transmission of these microorganisms in clinical cases [6]. *Yersinia pestis* is the causative agent of plague, a highly lethal zoonotic disease that wiped out a large swath of the European population during the Black Death of the Middle Ages. The evidence that *Y*. *pestis* is vectored and enzootically maintained by fleas, particularly species parasitizing rodents, is well-established and uncontroversial [7,8]. However, the evidence for body lice as vectors of plague bacilli is limited, making their involvement in the spread of the disease during previous plague pandemics highly controversial and a topic of great interest to historians, epidemiologists, entomologists, and microbiologists alike [9–11].

It is widely accepted that classic rat–rat flea–human transmission could account for the third plague pandemic, which began in Asia around 1860 and lasted until about 1920 [11–13]. However, a recent epidemiological modeling study that analyzed 9 plague outbreaks during the second pandemic, which included the Black Death of 1346–1353, indicated that a human ectoparasite mode of transmission (direct human–human transmission via body lice and/or the human flea, *Pulex irritans*) better matched the disease kinetics when compared to pneumonic or rodent flea transmission [11]. While *P*. *irritans* were likely abundant in households during the second pandemic, we have since reexamined the vector competence of the human flea and found it to be a poor vector of plague bacilli. In our analysis, *P*. *irritans* was highly inefficient at transmission and its proventriculus never became blocked by a *Y*. *pestis* biofilm, a condition that greatly enhances flea-borne transmission by causing the flea to feed excessively, when infected using human blood [14]. Existing data on human flea vector competence suggests that their importance in plague epidemiology is likely limited at best.

Few studies have directly evaluated the body louse’s ability to transmit *Y*. *pestis* in the laboratory setting, one using wild-type body lice collected from plague victims during an outbreak in Morocco [15], and the other (s) using a rabbit-adapted strain of body lice [16,17]. In the first instance, French physicians Blanc and Baltazard collected lice from plague victims and maintained them in the lab on convalescent humans or macaques for 0 to 8 days prior to challenging immunologically naïve guinea pigs [15]. In 10 experiments, where groups of 24 to 160 potentially infectious body lice fed to repletion after placement on the animals, only a single guinea pig developed plague, a 10% transmission rate. Following the experiments, the remaining lice were collected, macerated, pooled, and injected into rats or guinea pigs that subsequently developed plague, confirming that some lice had been colonized by *Y*. *pestis* for each of the experiments. More recently, a rabbit infection model was developed where the rabbit-adapted Orlando strain [18] of body lice were infected by feeding on rabbits after they had been intravenously inoculated with a high dose (1 × 10^9^ CFU) of *Y*. *pestis* 1 h prior [16]. After feeding on artificially bacteremic rabbits, groups of 10 to 300 lice were removed and transferred to naïve rabbits to feed daily. In 8 experiments, 7 of the 8 rabbits became infected with and died of plague, an 88% transmission rate. Orlando strain lice died rapidly following infection with *Y*. *pestis*, 25% to 50% within the first 48 h and 80% to 95% within 4 days of infection, significantly limiting their potential to vector *Y*. *pestis* to a narrow timeframe [16]. This rapid and high rate of louse mortality was not observed in the infected wild-type lice collected by Blanc and Baltazard [15]. The Orlando strain of lice, sometimes termed the Culpepper strain, has been feeding on rabbits since George Culpepper adapted them to do so roughly 80 years ago [18,19]. Thus, this strain is not as representative of wild lice as they have been conditioned to feed only once per day, with some derivations as infrequently as 3 times per week [20], and have probably evolved to process rabbit blood more efficiently than human blood as part of their continued adaptation to rabbits.

Collectively, these experiments demonstrated that body lice can become infected with and vector *Y*. *pestis* under optimal infection parameters but disagree substantially on the efficiency of body louse transmission and the duration of louse survival following *Y*. *pestis* infection. Furthermore, many key factors necessary for evaluating the body louse’s potential as a vector of *Y*. *pestis* remain to be determined, such as the minimum dose required to produce infective lice, the subsequent rate of chronic infection, the extrinsic incubation period, the louse organs that are colonized by *Y*. *pestis*, and the mechanism of transmission.

Here, we use a standardized membrane feeder-adapted body louse model to systematically analyze louse vector competence for *Y*. *pestis*. Due to concerns about the biological relevance of the Orlando strain, our results are generated using the San Francisco strain of body lice [21], as they are similar to wild lice that feed multiple times per day on human blood. Our analysis revealed that body lice become chronically infected, shed culturable *Y*. *pestis*, and routinely transmit plague bacilli following ingestion of infectious doses corresponding to the range of bacteremia observed in clinical cases of plague. Furthermore, using fluorescence microscopy and vector transmission assays we show that plague bacilli can colonize a set of putative salivary glands unique to lice, resulting in an enhanced ability of these lice to transmit by a previously undescribed bite-based mechanism.

## Results and discussion

### Simulating natural body louse infection scenarios

**Fig 1:**
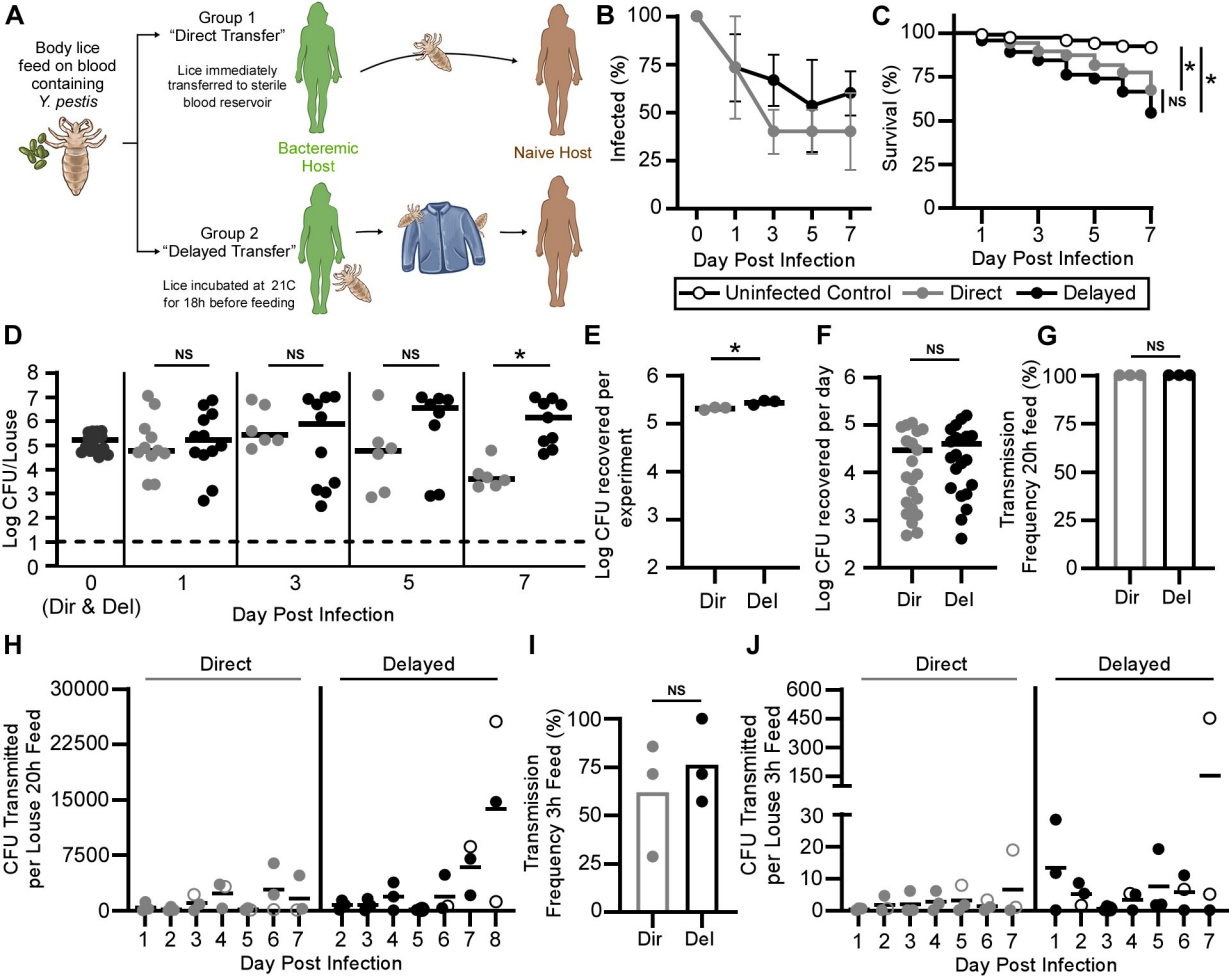
*Y*. *pestis* can be vectored by human body lice for at least 1 week following a single infectious blood meal. **(A)** Diagram of experimental protocols following infection of lice with *Y*. *pestis* to simulate the conditions following direct or delayed, fomite-based transfer of potentially infectious body lice between hosts. **(B)** Infection rate, **(C)** survival, and **(D)** bacterial burden over a 1-week period for groups of body lice fed on blood containing 3.5–5.8 × 10^8^ CFU/ml KIM6+(pmCherry) *Y*. *pestis*. Lice were allowed to begin feeding on sterile blood either immediately after infection (Direct) or 18 h later (Delayed). Data are pooled results from 3 independent experiments with *n* = 40 lice. For B and D, values are determined from 5 lice per experiment and analyzed using Fisher’s exact (B) or Mann–Whitney test; *p < 0.05 (D). Horizontal bars represent the median, dashed line shows the limit of detection, and day 0 values are identical for both infected louse groups. For C, *p < 0.0001 by Log-rank test. The cumulative number of *Y*. *pestis* CFU recovered from the feeding capsule blood reservoir for all transmission trials **(E)**; and for the individual daily trials **(F)** for each of the 3 experiments (*n* = 21). Horizontal bars represent the mean and *p < 0.05 by Student’s *t* test. **(G)** The percentage of days where transmission occurred and **(H)** the number of *Y*. *pestis* CFU recovered from the feeding capsule blood reservoir for the direct and delayed group lice after the 20-h feeding periods and after the shorter 3-h feeding periods **(I, J)**. Horizontal bars represent the mean and CFU values are normalized to the number of lice. Symbols represent results from 1 experiment and open circles signify instances where transmission occurred, but no *Y*. *pestis* was cultured from the feces. For G and I, data analyzed using Fisher’s exact test. For H and J, data analyzed using Student’s *t* test. Summary data for this figure can be found in S1 Data.

To investigate body louse vector competence, we decided to compare 2 scenarios that simulate how humans would be exposed to infected lice during a plague outbreak (Fig 1A). In the first “direct transfer” scenario, a human acquires infected lice from close contact with another human with a high-level *Y*. *pestis* bacteremia. Lice in this group were allowed to feed on human blood containing approximately 10^8^ CFU/ml *Y*. *pestis* KIM6+(pmCherry) for 1 h and then immediately transferred to a sterile feeding capsule where they could feed as desired. In the “delayed transfer” scenario, infected lice are acquired later from clothing, bedding, or from a human who has succumbed to septicemic plague. To simulate this off-host intermediary period, infected lice were held at a lower temperature (21°C) for 18 h, without access to blood, prior to being placed in a sterile feeding capsule. Body lice are highly susceptible to dehydration and generally do not survive well away from the host, typically dying within 2 to 4 days without a blood meal [1,22]. Following infection, body louse mortality, *Y*. *pestis* infection rate, transmission to the blood reservoir, fecal shedding, and localization within the louse were tracked for both groups for 1 week via culturing on agar media and fluorescence microscopy. Feeding capsules were changed twice a day, once after 20 h of continuous feeding and again 3 h later to quantitate bacterial transmission and fecal shedding for both extended and short feeding periods. Body lice are estimated to feed once every 4 h, so the short feeding period would be predicted to capture transmission occurring during a single feeding attempt by each infected louse in the capsule [1]. For each experiment, louse mortality was compared to a separate group of uninfected control lice that fed on sterile blood.

For both direct and delayed groups, 40% to 60% of the body lice remained infected for 1 week, indicating that body lice can develop a chronic *Y*. *pestis* infection following a high-dose infectious blood meal (Fig 1B). The delayed group tended to have higher numbers of infected lice, with the greatest difference occurring on day 3 (67% versus 40%); however, this difference was not statistically significant. For lice from both infection protocols, the 1-week mortality rate was significantly greater (33% to 46%) than the uninfected controls (8%) but did not differ significantly from one another (Fig 1C). On day 1 post-infection, the median bacterial burden for both direct and delayed groups was approximately 1 × 10^5^ CFU and bacterial burdens were similar between groups for day 3 and day 5. However, by day 7, the delayed group had a significantly higher median bacterial burden (approximately 1 × 10^6^ CFU) than the direct group (4 × 10^3^ CFU; Fig 1D).

For each of the 3 experiments, the cumulative number of bacteria recovered from the blood meal reservoirs over the entirety of the 7-day period, including both the 20 h and 3 h transmission assays, was modestly but significantly higher for the delayed group, on average 2.1 × 10^5^ CFU for the direct group lice and 2.8 × 10^5^ CFU for the delayed group (Fig 1E). Distribution of the cumulative CFUs recovered (Average = approximately 3 × 10^4^ CFU) from the daily transmission assays was more or less identical for both scenarios across experiments (Fig 1F).

During the 20-h feedings, transmission was observed in 100% of the assays for both infected louse groups (Fig 1G). *Y*. *pestis* CFUs transmitted and shed in the feces were similar for both louse acquisition scenarios, ranging from 12 to 25,600 CFU transmitted per louse and 0 to 7,083 CFU defecated per louse (Figs 1H and S1A). The number of CFU recovered after the longer 20-h transmission assays likely reflects some bacterial reproduction in the blood reservoir following transmission from lice. This feature of the 20-h feeding assay was beneficial for observing the potential occurrence of low-level, inefficient, and infrequent transmission events but makes differences in the number of bacteria recovered more difficult to assign significance to.

Transmission of *Y*. *pestis* by body lice was also frequently observed during the subsequent 3-h feeding periods across all 3 experiments, with 62% to 76% of the feedings resulting in recovery of culturable bacteria from the blood reservoir (Fig 1I). During the short 3-h feedings for direct transfer lice, a range of 0 to 284 CFU were recovered from the blood reservoirs fed upon by 15 to 40 lice or 0 to 19 CFU transmitted per louse (Fig 1J). Comparatively, delayed-group lice yielded higher maximal CFU values transmitted (range = 0–4,512 CFU, or 0–451 CFU per louse). Bacteria were routinely recovered from feces deposited by the lice on the surface of the parafilm feeding membrane for both louse groups, with values ranging from 0 to 1,522 CFU defecated per louse (S1B Fig). The number of bacteria recovered from feces was similar for both direct and delayed infected louse groups (S1 Fig). Unexpectedly, for both the longer 20 h (9 of 42 assays) and shorter, 3 h feedings (11 of 29 assays), transmission regularly occurred when no bacteria could be recovered and cultured from feces at the conclusion of the feeding period. These events were observed at least once for both infection scenarios in all 3 experiments (open symbols; Fig 1H and 1J).

Overall, differences between the 2 infected louse-acquisition scenarios were modest. The delayed group typically had slightly higher infection, mortality, transmission rates, and maximal numbers of CFU transmitted (Fig 1). However, for all of the parameters assayed, differences usually did not reach statistical significance when compared to the direct transfer louse group. Collectively, the data suggest that infected lice that spend a short time away from their host (Delayed scenario; Fig 1A) are modestly more susceptible to chronic *Y*. *pestis* infection and are more effective vectors. However, infected lice from both acquisition scenarios routinely became infected with and transmitted *Y*. *pestis*. Notably, the extrinsic incubation period, or the time required for a vector to become infectious after acquiring the pathogen, is very short for body lice under these experimental conditions, as lice from the direct-transfer scenario began transmitting bacteria within the first 24 h of infection (Fig 1).

Even though fewer infected lice survived in the feeding capsules as time progressed due to *Y*. *pestis*-induced mortality (Fig 1B), the overall rate and efficiency of transmission remained stable or slightly improved by day 7 post-infection for both groups (Fig 1H and 1J), suggesting that the remaining infected lice had an enhanced capacity to transmit *Y*. *pestis*. Furthermore, transmission of most louse-borne bacterial pathogens occurs primarily through contamination of the wound or bite-site with feces or crushed insects, so the absence of bacteria in the feces for 21% to 38% of the positive transmission assays (Fig 1H and 1J) was unanticipated. Collectively, these data suggested a body louse transmission mechanism independent of blood reservoir inoculation with *Y*. *pestis*-laden fecal material.

### *Y*. *pestis* colonizes a unique set of putative salivary glands in a subset of infected lice

**Fig 2:**
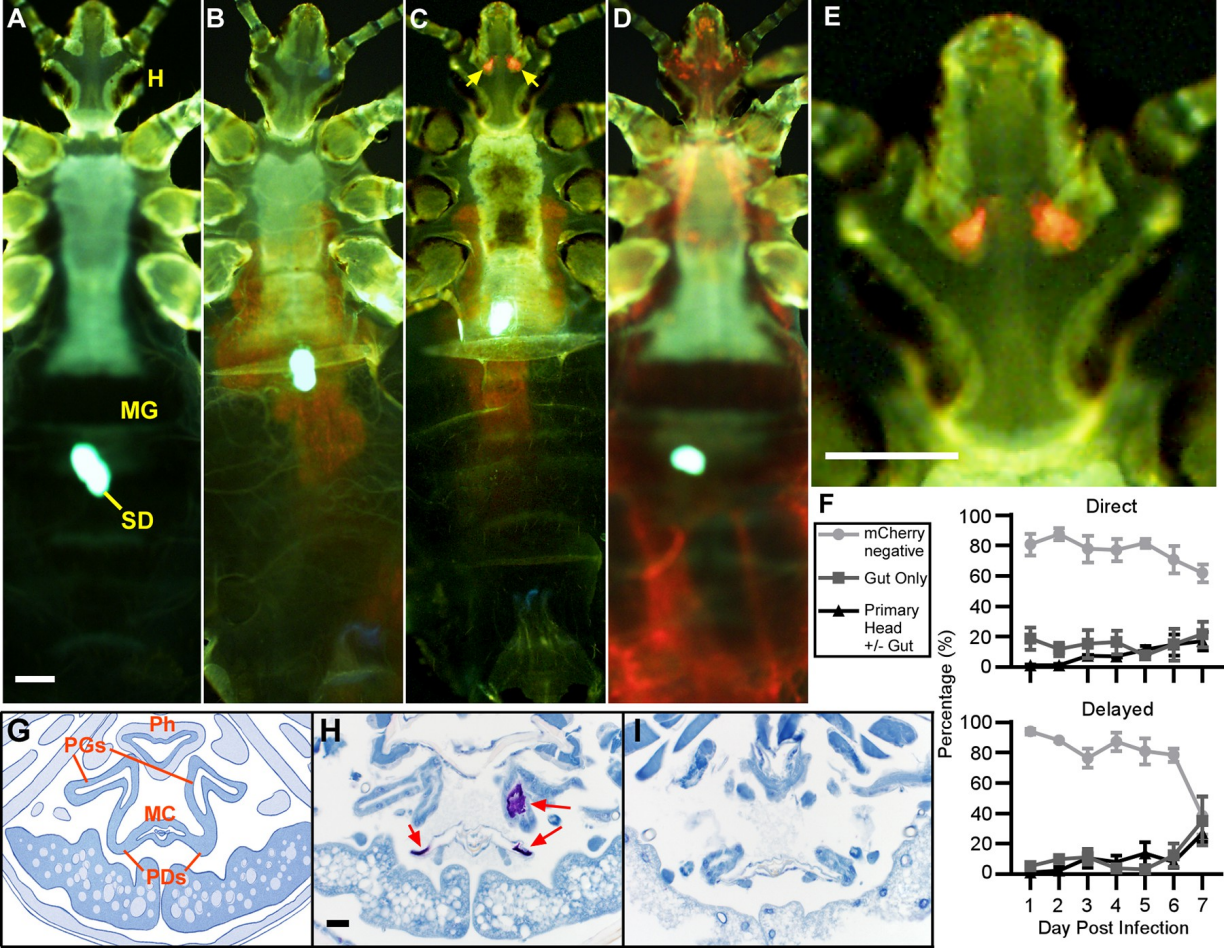
*Y*. *pestis* can colonize the Pawlowsky glands, a set of putative salivary glands in the louse head. Fluorescence microscopy images of KIM6+(pmCherry) *Y*. *pestis* localization patterns observed in body lice following infection: **(A)** mCherry (orange or red) negative (no infection detected) **(B)** midgut only infection (**C)** 1 or 2 foci of infection in the head (yellow arrows; with or without bacteria simultaneously detected in the midgut) or **(D)** bacteremic (bacteria detected in the hemocoel). **(E)** Cropped and enlarged image of the head of infected louse in panel C. MG = Midgut, H = Head, SD = Stomach disc. The louse exoskeleton and SD are autofluorescent. Scale bars = 200 μm. **(F)** The frequency of infection patterns observed (*n* = 35–40 lice) from the direct (top) or delayed (bottom) groups for 1 week of infection. Vertical bars show the standard error of the mean for 3 independent experiments. **(G)** Diagram of a transverse section of the head of a body louse based on our histology and [23]. Major anatomical structures include: the pharynx (Ph), the Pawlowsky glands (PGs), the PG ducts (PDs), and the chamber housing the mouthparts (MC). **(H)** Immunohistochemical detection of *Y*. *pestis* infection (red arrows; purple/pink staining) in the PGs and ducts that lead to the MC from a louse diagnosed with foci of infection in the head. Scale bar = 20 μm. **(I)** Absence of *Y*. *pestis* staining in an uninfected control louse. Images are representative of 5 μm sections of interest acquired from 4 infected and control lice. IHC images are counterstained with hematoxylin (blue). Summary data for this figure can be found in S2 Data.

To determine the structures that *Y*. *pestis* colonizes in body lice and give insight into possible transmission mechanisms, we screened whole infected lice for *Y*. *pestis* engineered to express mCherry using fluorescence microscopy. During fluorescence screening we observed that infected lice typically displayed 1 of 3 *Y*. *pestis* localization patterns: (1) lice with no detectable bacteria within any louse structure (Fig 2A; mCherry negative); (2) lice with bacteria observed only in the midgut (Fig 2B; gut-only infection); or (3) lice with 1 or 2 typically ovular-shaped foci of bacteria located in the head, with or without the additional presence of bacteria in the gut (Fig 2C and 2E; primary head infection). Occasionally (6 out of 240 lice across all experiments), lice were observed with bacteria throughout the entire body, indicating the infection can infrequently spread into the hemocoel prior to death (Fig 2D). These bacteremic lice were typically red-colored and moribund: lethargic, unable to right themselves, and frequently died during imaging. For most of the 1-week infection period, the majority of lice (approximately 75% to 85%) in both infection scenarios did not have detectable mCherry signal, though these percentages decreased to approximately 40% to 60% by day 7 (Fig 2F). The ~15% to 25% of lice with detectable bacteria were split between the gut-only and primary head infection patterns. In total, 14% of the infected lice screened, at least 33 of 240 unique individuals from both infection scenarios, developed a head infection over the 7-day period. In one experiment, head infection was observed as early as 1 day after infection; however, they typically were not observed until day 3 post-infection or later. This was unexpected, as we are unaware of any microbial infections localizing to structures within the head of the louse. In contrast, the percentage of lice with a gut-only infection was fairly stable throughout the assays.

Body lice have 2 different sets of salivary glands: distinct pairs of u-shaped and bean-shaped sets located within the thorax, and a third set of putative salivary glands, termed the Pawlowsky glands (PGs), located within the head [23–25]. The biological function of the PGs is not entirely clear and, as such, their status as salivary glands is tenuous. However, these glands are attached to narrow ducts that feed into the chamber housing the body louse’s retractable piercing-sucking mouthparts [23,25]. Based on these and other observations, the PGs are speculated to produce lubricant to aid in mouthpart extension and retraction during blood feeding [25]. The ovular shape and location of the fluorescent signal(s) from lice with a *Y*. *pestis* head infection are consistent with the description of the PGs in anatomical illustrations of body lice (Fig 2C, 2E, and 2G). To verify that PGs were the structures becoming infected with *Y*. *pestis*, we performed immunohistochemistry (IHC) on transverse sections of fixed, uninfected control lice or lice diagnosed with a head infection (Fig 2H and 2I). *Y*. *pestis* was detected in the PGs, within their ducts that intersect with the mouthpart housing chamber, or within both structures in sections from 4 lice diagnosed with fluorescent foci of infection within the head (Fig 2H), while no staining was detected in the sections acquired from uninfected control lice (Fig 2I).

### Lice with infected Pawlowsky glands routinely transmit *Y*. *pestis* during blood feeding

**Fig 3:**
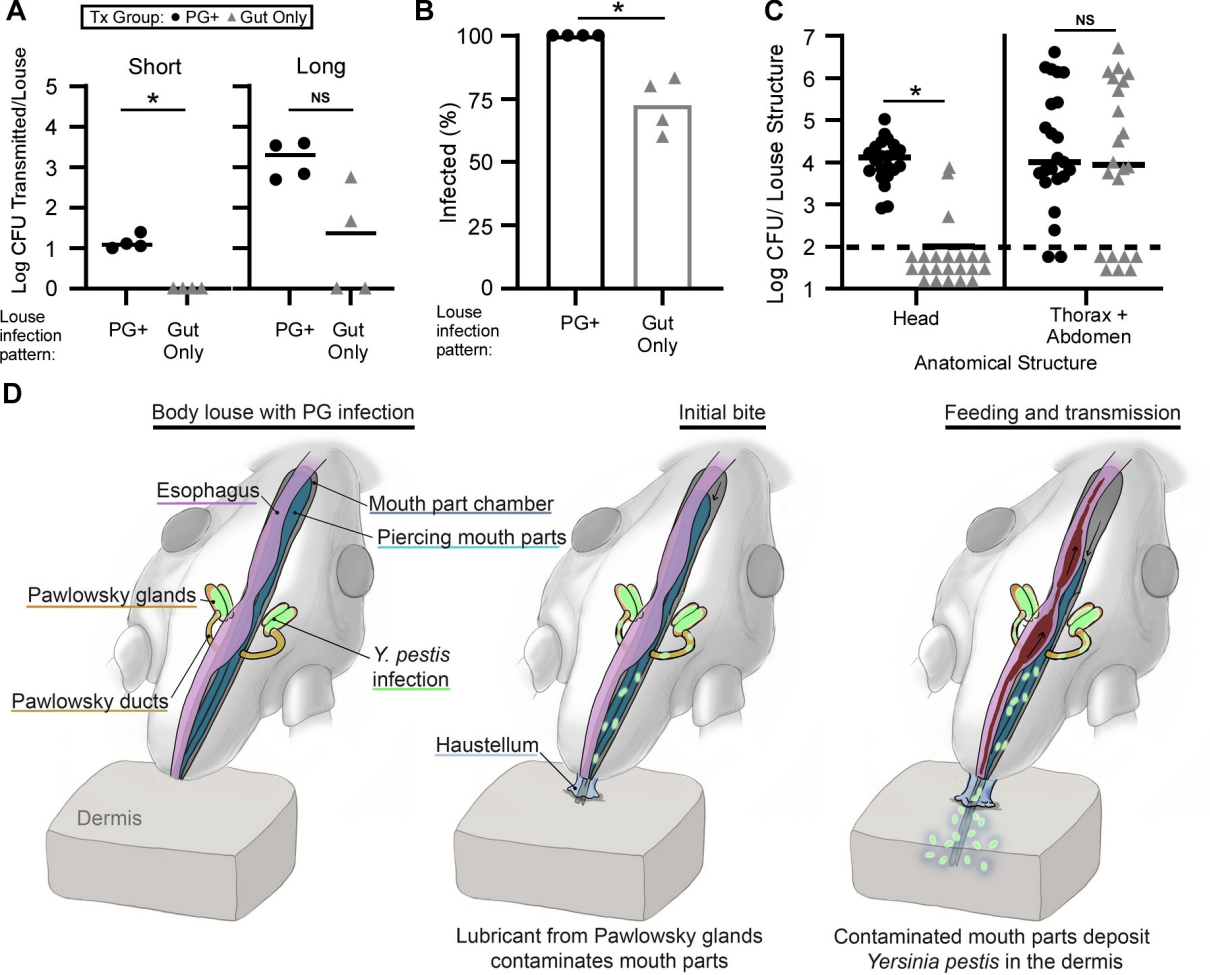
Lice with infected Pawlowsky glands routinely transmit *Y*. *pestis* by a bite-based mechanism. Lice were fed on blood containing 1.4–4.1 × 10^8^ CFU/ml KIM6+(pmCherry) *Y*. *pestis* and 6 days later groups of 5–6 lice diagnosed with a Pawlowsky gland infection (PG+) or a midgut-only infection (Gut only) were assayed for their ability to **(A)** transmit KIM6+ (pmCherry) *Y*. *pestis* during a 20 h (long) and subsequent 3 h (short) feeding period. **(B)** The percentage infected and **(C)** bacterial burden present in the head or thorax and abdomen of lice at the end of the transmission assays. Data are pooled results from 4 independent experiments with 22–23 lice total (*n* = 5–6 per experiment). Dashed line represents the limit of detection. For A and C, horizontal bars represent the median and *p < 0.05 by Mann–Whitney test. For B, bars show the average and *p < 0.01 by Fisher’s exact test. **(D)** Model of *Y*. *pestis* transmission by human body lice with Pawlowsky gland infection. Secretions from the infected PGs mixed with *Y*. *pestis* (green) contaminate the body louse mouthparts prior to blood feeding. Once the louse anchors itself to the host skin with its haustellum, the contaminated mouthparts are extended, puncture the skin, and deposit *Y*. *pestis* into the host dermis during routine blood ingestion. Summary data for this figure can be found in S3 Data.

Next, we determined if lice with a PG infection were more effective at transmitting *Y*. *pestis* than those that had bacteria present only in the midgut. Groups of infected lice were screened using fluorescence to diagnose either a primary PG infection or a midgut-only infection and groups of 5 to 6 with identical diagnoses were isolated. Infected louse groups were then transferred to a sterile feeding chamber and transmission of *Y*. *pestis* to the blood reservoir was monitored after both a long (20 h) and subsequent short (3 h) feeding period. During the short 3-h feeding period, in 4 independent experiments, transmission was only detected for lice with a PG infection (Median = 68 CFU recovered, 12 CFU/louse) while no transmission was detected for groups with a midgut-only infection (Fig 3A). Again, during the longer 20-h feeding assays, transmission was always detected for lice with a PG infection but was detected in only half the experiments for those with infection observed only in the midgut. The number of CFU recovered per louse for the longer transmission assays in the PG infection group (Median = 2 × 10^3^ CFU/louse) was higher than the midgut only group (Median = 23 CFU/louse).

At the end of the transmission experiments and to confirm our infection pattern diagnoses, body lice were collected, surface sterilized, the head was separated from the thorax and abdomen using scissors, and both louse segments were plated on agar medium to screen for infection. All of the lice from the PG+ groups remained infected by the end of the assay, but only approximately 75% from the gut-only group (Fig 3B), indicating that lice with bacteria present only in the midgut more readily clear the infection during routine feeding than those with PG infection. *Y*. *pestis* was recovered from the head of all of the lice diagnosed with PG infection (Median = 1.3 × 10^4^ CFU). In contrast, only 3 of 22 lice diagnosed with a gut-only infection had *Y*. *pestis* present within the head (Fig 3C). We suspect the infection present in the PGs of these lice was below the limit of detection of fluorescence microscopy screening, causing them to be misdiagnosed. Two of these false-negative, PG-infected lice were detected in one of the experiments where transmission occurred during the longer transmission assay, and we think it likely these were the lice responsible for *Y*. *pestis* transmission (Fig 3A). However, both the PG+ and midgut-only infected louse groups had similar bacterial burdens present in the abdomen and thorax (Median = approximately 1 × 10^4^ CFU), indicating that the distinguishing feature of lice with enhanced transmission potential was the presence of a significant *Y*. *pestis* infection within the PGs.

Our experiments demonstrate that *Y*. *pestis* can colonize human body lice and be transmitted for at least 1 week, and likely longer (Figs 1–3). Of particular import is the finding that the body louse PGs can become colonized by *Y*. *pestis* in a subset of infected lice which routinely transmit plague bacilli in concentrations sufficient to initiate disease in humans (Figs 2 and 3A–3C). We propose a model (Fig 3D) in which *Y*. *pestis* colonizes the PGs immediately after the initial infectious blood meal, upon retraction of mouthparts contaminated with infectious blood. The ducts of the PGs slope modestly downward from the chamber that houses the mouthparts (Figs 2G–2I and 3D). When the lice finish feeding, residual infected blood present on the mouthparts may pool near the base of the ducts where the bacteria begin to reproduce (Fig 2H). Over the course of a few days, via fluid exchange and bacterial expansion, the infection spreads from the ducts to the glands, often reaching concentrations detectable with fluorescence microscopy 3 to 5 days after initial infection (Fig 2F). We hypothesize that *Y*. *pestis*-laden secretions from infected PGs pass through the ducts and repeatedly coat the body louse’s piercing-sucking mouthparts prior to each feed (Fig 3D). Upon anchoring itself to the skin using its haustellum, equipped with protruding epipharyngeal teeth [25,26], the body louse extends its mouthparts, pierces the skin, and deposits *Y*. *pestis* into the host dermis during each subsequent blood meal.

Collectively, the data indicate that lice that develop a PG infection transmit *Y*. *pestis* more consistently and in greater numbers than those with solely a midgut infection in our transmission model.

### The incidence of Pawlowsky gland infection correlates with infectious dose

In our initial experiments (Fig 1), and as proof of concept for body lice as potential vectors of *Y*. *pestis*, lice fed on blood containing ≥10^8^ *Y*. *pestis*/ml, which may not reflect physiologically relevant concentrations of human bacteremia during natural plague infection. Therefore, we tested a wider range of bacterial concentrations in the infectious blood that encapsulate lower human plague bacteremia levels (10^4^–10^7^ CFU/ml) using the delayed-transfer protocol (Fig 1A). We determined that the minimum bacteremia level required for *Y*. *pestis* to reliably cause chronic (up to 1 week) infection in 10% to 20% of body lice and subsequent recurrent defecation of culturable plague bacilli was 1.2 × 10^5^ CFU/ml (Table 1). *Y*. *pestis* was cultured from a single louse sampled 1 week after infection at the lower bacteremia of 1.2 × 10^4^ CFU/ml; however, this was only observed in one of the 3 experiments. At the highest bacteremia tested (approximately 4 × 10^8^ CFU/ml), the average rate of infection after 7 days was 58%. The threshold level required to detect lice with a PG infection and subsequent bite-based transmission was 1.2 × 10^7^ *Y*. *pestis*/ml of blood (Table 1). For all experiments with lower bacterial concentrations, PG infection and transmission to the blood reservoir was not detected.

**Table 1:** Summary of body louse infection experiments with *Y*. *pestis* KIM6+(pmCherry).

Experiment #	*Y*. *pestis* CFU/ml in the infectious blood meal	Total # of lice (*n*)	Infection rate (day 7 post-infection)	*Y*. *pestis* cultured from feces (days 3–7)	Transmission of *Y*. *pestis* (days 3–7)	Detection of body lice with PG infection (days 3–7)
Bacteremia level >10^8^ CFU/ml						
1*	4.3 × 10^8^	40	40%	+	+	+
2*	5.8 × 10^8^	40	60%	+	+	+
3*	3.5 × 10^8^	40	80%	+	+	+
4	4.5 × 10^8^	46	71%	+	+	+
5	2.6 × 10^8^	44	40%	+	+	+
Average	**4.1** × **10**^**8**^	**42**	**58%**	**+**	**+**	**+**
Bacteremia level 10^7^–10^8^ CFU/ml						
1	2.2 × 10^7^	40	29%	-	+	+
2	1.3 × 10^7^	39	60%	+	+	+
3	2.3 × 10^7^	45	40%	+	+	+
Average	**1.9** × **10**^**7**^	**41**	**43%**	**+**	**+**	**+**
Bacteremia level 10^6^–10^7^ CFU/ml						
1	2.1 × 10^6^	38	60%	+	-	-
2	3.2 × 10^6^	41	20%	+	-	-
3	1.6 × 10^6^	40	22%	+	-	-
Average	**2.3** × **10**^**6**^	**40**	**34%**	**+**	**-**	**-**
Bacteremia level 10^5^–10^6^ CFU/ml						
1	1.4 × 10^5^	32	20%	+	-	-
2	2.4 × 10^5^	43	20%	+	-	-
3	1.2 × 10^5^	40	11%	+	-	-
Average	**1.7** × **10**^**5**^	**38**	**17%**	**+**	**-**	**-**
Bacteremia level 10^4^–10^5^ CFU/ml						
1	3.0 × 10^4^	43	0%	-	-	-
2	2.1 × 10^4^	38	0%	-	-	-
3	1.2 × 10^4^	40	11%	+	-	-
Average	**2.1** × **10**^**4**^	**40**	**4%**	**+/-**	**-**	**-**

* Data from experiments shown in Fig 1. All louse infections used the delayed transfer infection protocol (Fig 1A).

The maximal bacteremia reported in a plague patient is 4 × 10^7^ CFU/ml and was cultured at the time of patient admission by US Army physicians in Vietnam [27]; however, the majority of documented values from clinical cases involve plague bacilli concentrations in the blood approximately 10^6^ CFU/ml or less [28,29]. Although the bacteremia levels required to initiate PG infection (≥10^7^/ml) are at the upper end of the range reported for human bacteremia, it is important to put existing clinical data into context [27,29]. Plague bacteremia levels have typically been quantified at the time of hospital admission, when patients were in various stages of disease progression. As far as we are aware, no study has explicitly and exclusively quantified bacteremia levels during the terminal stage of septicemic plague. A distinguishing feature of plague in mice and rats, where bacteremia concentrations in the blood can exceed 10^8^ CFU/ml, is the rapid increase in bacterial blood concentrations, perhaps an order of magnitude or more, in the final hours before death [30–32]. This may be the case for humans as well, making it difficult to evaluate how frequently plague bacteremia reached the concentrations required to effectuate body louse PG infection during previous plague pandemics. Notably, reported blood volumes ingested by body lice (approximately 1 to 3 μl) are considerably larger than either rat or human fleas (approximately 0.25 to 0.5 μl); indicating they would be challenged with greater numbers of *Y*. *pestis* when taking an infectious blood meal [14,33–35]. Interestingly, human body lice and head lice are ecotypes of the same species (*Pediculus humanus*) that preferentially parasitize different regions of the body [36,37]. Head lice are not known to or believed to vector any pathogens, possibly due to the comparatively smaller volumes of blood ingested and more frequent feeding relative to body lice [38].

Blood feeding by body lice induces a strong itching response in humans [1]. The resultant scratching can cause skin abrasions and provide an entry point for bacteria-laden louse feces or contaminated fluids from crushed lice. Even for body lice that fed on blood containing relatively low levels of *Y*. *pestis* (approximately 10^5^ CFU/ml), we routinely cultured bacteria from louse feces (Table 1). Because *Y*. *pestis* is so virulent and the fact that lice feed and defecate so frequently, it is possible that fecal transmission could occur even in instances where the infectious dose was not sufficient to initiate PG infection. In natural body louse infestations, *Y*. *pestis*-laden feces and perhaps even more likely, fluids released from crushed lice, could potentially enter the host through an abrasion generated during scratching. Furthermore, because body lice feed several times per day, they could potentially ingest multiple infectious blood meals from a bacteremic human, plausibly increasing the overall infection rate and incidence of PG colonization.

The data indicate that chronic infection of the body louse midgut occurs within a range of bacteremia routinely observed in clinical case reports, while infection of the PGs can result at the upper range of reported human plague bacteremia [10,27].

### Ability to infect the body louse Pawlowsky glands is not universal among gram-negative bacteria

Human body lice lack key genes in the arthropod innate immune cascade (IMD) that would allow them to produce effector molecules against gram-negative bacteria [39]. Likely as a result, body lice are believed to be more, if not broadly, susceptible to infection by these microorganisms. As such, we wanted to determine if the ability to infect body lice and be transmitted was a trait generalizable to the *Enterobacteriaceae* or limited to *Yersinia* spp. or to certain *Y*. *pestis* biovars. Furthermore, *Y*. *pestis* evolved from the mild enteric pathogen *Yersinia pseudotuberculosis* to become a highly virulent flea-borne pathogen within the last 5,000 years [40,41]. Notably, only a handful of genetic changes facilitated the bacterium’s transition to a flea-borne life cycle [8], and we wanted to determine whether 2 key genetic elements that facilitate flea midgut colonization and efficient transmission from the flea foregut (*ymt* and *hmsHFRS*, respectively) were also critical for body louse infection.

In addition to *Y*. *pestis* KIM6+ (Biovar Medievalis), we infected body lice using blood containing a high dose (>10^8^ CFU/ml) of (1) the Biovar Orientalis strain *Y*. *pestis* CO92 (pCD^–^); (2) a *Y*. *pestis* KIM6+ Ymt mutant (defective for flea midgut colonization; [35,42]); (3) a *Y*. *pestis* KIM6+ Hms mutant (defective for flea blockage and transmission; [43]); (4) *Yersinia pseudotuberculosis* IP32953 (the evolutionary progenitor of *Y*. *pestis* [44]); or (5) *Escherichia coli* DH5α (a common laboratory molecular cloning strain). Ultimately, all 6 of the bacterial strains assayed were capable of stably colonizing body lice (S2A Fig). The 4 *Y*. *pestis* strains infected 40% to 70% of lice for up to 1 week, including the Ymt-negative mutant that is typically eliminated from the gut of fleas within 24 h of an infectious blood meal [42]. Both *Y*. *pseudotuberculosis* and *E*. *coli* had a higher body louse infection rate (70% to 100%) and bacterial burden than *Y*. *pestis* (S2A and S2B Fig).

All of the bacterial strains tested were capable of infecting the louse midgut and bacteria were routinely cultured from the feces of these lice throughout the 1-week infection period (S2C and S2E Fig). Notably, only *Yersinia* spp. (and not *E*. *coli*) were capable of infecting the body louse PGs. Furthermore, *E*. *coli* DH5α was never transmitted by lice to the blood reservoir (S2D and S2F Fig). In contrast, all of the *Y*. *pestis* strains tested had roughly equivalent rates of PG infection and routinely transmitted plague bacilli (S2D and S2F Fig). Interestingly, *Y*. *pseudotuberculosis* colonized the PGs at a higher rate yet was transmitted less frequently and in noticeably lower amounts when compared to the *Y*. *pestis* strains (S2D and S2F Fig). Infected body louse mortality rates were equivalent for all the bacterial strains tested except *Y*. *pseudotuberculosis*, which caused about twice as much mortality (S3A Fig). Unlike infections with the other bacterial species, lice infected with *Y*. *pseudotuberculosis* frequently turned red shortly before death; suggesting that their midgut had been damaged, allowing some of the blood meal to leak into the hemocoel (S3B Fig). These observations of high mortality are consistent with a previous study that monitored *Y*. *pseudotuberculosis* infection kinetics in body lice infected intrarectally [45].

Consistent with previous reports, the body louse midgut was highly susceptible to infection by gram-negative bacteria in our model. Interestingly, the bacterial strains differed in their ability to colonize the PGs and be transmitted by lice (S2 Fig). Two different biovars of *Y*. *pestis* could routinely infect the PGs in a subset of the infected lice, conflicting with a previous report showing resistance of Orlando strain body lice to colonization with Biovar Medievalis *Y*. *pestis* after feeding on infected rabbits [17], whereas *E*. *coli* DH5α was incapable of colonizing the glands and being transmitted. Colonization of the PGs or transmission from the louse was not dependent on expression of the *Y*. *pestis* Ymt phospholipase D enzyme (flea midgut colonization) or the extracellular polysaccharide product of the Hms operon (flea biofilm and blockage formation), which are the 2 most important genetic elements for the *Y*. *pestis* life stage in fleas [46] (S2 Fig). *Y*. *pseudotuberculosis*, the evolutionary progenitor of *Y*. *pestis*, is known to be more adherent and invasive to epithelial tissues than *Y*. *pestis* [47], mediated partly by the invasin gene [48], which is a pseudogene in *Y*. *pestis* [49,50]. This may account for its greater infectivity to lice, as well as less-efficient transmission by our model if it is more adherent to the PGs and its ducts (S2A, S2D, S2F, and S3 Figs) [45]. It remains to be determined if there is some degree of evolutionary adaptation of *Y*. *pestis* to body lice and whether specific genetic elements facilitate colonization and transmission from these glands.

### Summary and Conclusion

In summation, key features of human body lice that likely contribute to their ability to vector *Yersinia pestis* include: (1) They ingest a larger volume of blood than fleas in a single feeding, increasing the number of bacteria they are challenged with during an infectious blood meal and likely reducing the bacteremia threshold necessary to initiate infection. (2) Body louse frequent feeding behavior increases the chances that lice developing a Pawlowsky gland infection will transmit plague bacilli in sufficient cumulative numbers to cause disease. (3) Body lice lack key arthropod immunity genes that are likely important in moderating gram-negative bacterial infections in the midgut [39], resulting in frequent defecation of virulent organisms. In a natural setting, the pruritis effectuated by their bites and the resultant scratching generates abrasions that can serve as an entry point for pathogens such as *Y*. *pestis*.

Human plague outbreaks during the Black Death tended to have a strong seasonal pattern, peaking from late spring to early fall, correlating well with climatic conditions when rodent fleas are most abundant [13,31,51]. Blanc and Baltazard and others have proposed that human plague outbreaks are dependent on and originate from a rodent-flea driven epizootic focus, but that epidemic spread could then be significantly fueled by transmission via human ectoparasites [9,11,15,52]. Our results suggest body lice are better vectors of plague bacilli than previously appreciated and add support to the hypothesis that body lice were contributing vectors in previous human outbreaks under certain environmental and ecological conditions.

## Methods

### Body louse colony maintenance and feeding

The San Francisco strain of human body louse (*Pediculus humanus humanus*), founded by Dr. John Clark (University of Massachusetts-Amherst), was established at the Rocky Mountain Laboratories using previously described methodology with minor modification [21]. San Francisco body lice were housed in capsules constructed from truncated 50 ml conical tubes (Celltreat Scientific Products) and affixed with a silicone-parafilm M sandwich membrane. The upper portion of the capsules were constructed by sawing conical tubes at the 35 ml marker (retaining the threaded end and lid), applying a nickel-sized volume of silicone aquarium sealant on 2 × 4 inch strips of parafilm M, folding the parafilm in half, flattening the sandwich membrane with a rolling device to remove excess silicone, stretching the parafilm roughly twice its original size, wrapping it over and around the threaded end of the tube, and trimming the excess membrane. Capsule membranes were cured for at least 24 h prior to use. To prepare louse-ready feeding capsules, a 1.5 × 0.1 × 0.1 cm magnetic stir bar was placed in the lid of the conical tubes and surface sterilized with 70% ethanol. Next, 3 ml of reconstituted or whole citrated human blood (Innovative Research; 3.8% sodium citrate; FDA approval #3003372368), supplemented with 50 units of penicillin and 50 μg of streptomycin per ml, was added to the lid. Finally, the upper portion of the capsule was screwed into the newly created blood reservoir (roughly ¼ turn when the threads of the tube and lid are aligned) to maximize contact with the blood, eliminate air bubbles, and allow sufficient space for the stir bar to rotate. Lice on custom approximately 1.0 × 0.5 × 0.5 inch black synthetic hair tufts (glued together at one end with silicone) were placed within the inner chamber of the feeding capsules on top of the membrane, allowing them to feed from above. Feeding capsules are placed on the ports of a multipoint-15 CIMAREC i magnetic stirrer (Thermo Scientific) to prevent red blood cell sedimentation (150 RPM) and stored within a humidified environmental chamber (30°C, 60% RH) overnight. Hair tufts containing lice were transferred to a fresh capsule daily. Stir bars were cleaned and sterilized prior to reuse.

### Body louse infection

*Yersinia pestis*, *Yersinia pseudotuberculosis*, or *Escherichia coli* strains (S1 Table) transformed with a plasmid constitutively expressing the mCherry red fluorescent protein (pMcherry; Takara Bio) were streaked for isolation on blood agar plates (5% sheep’s blood), incubated for 24 (*E*.*coli* and *Y*. *pseudotuberculosis*) or 48 h (*Y*. *pestis*) at 28°C, and a single large colony was selected to inoculate 100 ml of brain heart infusion (BHI) broth supplemented with 10 μg/ml hemin and 100 μg/ml carbenicillin. Cultures were then incubated for 18 to 19 h at 37°C. A portion of the bacterial culture, based on optical density, was centrifuged at 6,000 rpm for 10 min and bacteria were resuspended in 0.5 ml of sterile PBS and added to 2.5 ml of citrated human blood supplemented with 100 μg/ml carbenicillin. Serial dilutions of the infectious blood meal were plated on sheep blood agar/carbenicillin plates to determine the bacteremia level.

Prior to oral infection, age-matched adult body lice, hatched 2 to 3 weeks prior and approximately equal numbers male and female, were transferred to a fresh hair tuft and allowed to feed on blood supplemented with carbenicillin for 8 h. Following this preinfection feeding period to eliminate residual streptomycin, lice were starved for 16 to 18 h overnight where the insects purge the majority of their stomach contents via defecation.

Starved lice were then fed for 1 h on infectious blood and screened visually for evidence of feeding using a dissection microscope. A sample of 5 to 10 infected lice was immediately frozen at −80°C for later determination of the infectious dose.

Following infection, lice were separated into 1 of 2 feeding schedule groups: direct or delayed transfer. For the direct group, lice were immediately transferred to a fresh, sterile capsule containing blood supplemented with carbenicillin so that louse feeding could resume within the humidified incubator. For the delayed group, lice underwent a second, post-infection 18-h starvation period in a humidified chamber (21°C, 70% RH) prior to initiating sterile blood feeding as described above. Feeding capsules were replaced twice a day, every day thereafter at 2 intervals: once after an extended 20-h overnight feeding period and again after a shorter 3-h feeding period. These feeding durations were designed to determine the CFU transmitted by lice during a single feeding event (body lice feed approximately 6 times a day, approximately once every 4 h), as well as general evidence of transmission and bacterial shedding in the feces during the longer 20-h feeding period, where a degree of post-transmission bacterial reproduction may occur within the blood reservoir.

For both groups of infected lice, the following was done daily: lice were counted to record mortality, examined by fluorescence microscopy to track infection localization patterns utilizing the mCherry protein expressed by the bacteria, blood from the capsule reservoirs was plated to track transmission, and the feces collected from the exterior surface of the feeding membrane. In addition, 5 lice were removed and frozen at −80°C on days 1, 3, 5, and 7 for later determination of *Y*. *pestis* concentrations in the lice. Because the delayed group lice did not resume feeding until 18 h after infection, data for the first 20-h transmission and fecal CFU assays were collected on day 2 post-infection instead of on day 1 as for the direct group. Post-infection times of all other assays and data collections were the same for the 2 groups.

For comparisons of body louse infection with different bacterial strains and infectious doses performed in Table 1 and S2 and S3 Figs, lice were infected using the delayed-group experimental protocol and transmission and fecal shedding was screened for days 3 to 7 postinfection; 5 to 10 lice were collected on days 3 and 7 for determination of *Y*. *pestis* bacterial burden and infection rate.

### Monitoring *Y*. *pestis* infection and transmission kinetics in lice

For fluorescent imaging, lice were placed in a petri dish on ice to immobilize them at the conclusion of the short 3-h feeding period. After 10 min, individual lice were transferred, ventral side up, to an ice-cold drop of PBS on a microscope slide using a pair of fine forceps. An 18 × 18 mm glass cover slip was gently placed over the top of the lice and additional PBS was added until lice were enveloped and immobilized. Fluorescent images of *Y*. *pestis* infection within the lice were obtained using a Nikon Eclipse E800 microscope and the ET-DAPI/FITC/TRITC fluorescent filter cube (Nikon). With this filter, autofluorescence from the body louse cuticle and stomach disc appears mint green in images. All pictures were obtained with an Olympus DP72 camera and cellSens software. For comparisons of body louse infection with different bacterial strains performed in S2 Fig, lice were stored in the humidified incubator, without access to blood, for 3 h prior to fluorescent imaging on days 3, 5, and 7.

To enumerate CFU recovered from the capsule blood reservoirs and louse feces, 1 ml of sterile PBS was added to the inner chamber of the capsules, swirled briefly by hand, and incubated at room temperature for 10 min. Next, resuspended feces were removed with a pipette, transferred to a 1.5 ml tube, briefly vortexed, and 10-fold serial dilutions of the mixture were plated on blood agar supplemented with carbenicillin. Next, the capsule was separated from the blood reservoir and blood was distributively plated on blood agar supplemented with carbenicillin to enumerate CFUs transmitted. A 10-fold serial dilution series of the blood was also plated.

For CFU determination in frozen louse samples, body lice were surface sterilized with 95% ethanol, transferred to a tube containing 1 ml of sterile PBS and lysing matrix Z beads (MP Biomedicals, Santa Anna, California), and mechanically disrupted with a bead beading apparatus. Dilutions of triturated lice were plated in BHI soft agar overlays supplemented with carbenicillin, hemin (*E*. *coli*), and irgasan (*Yersinia* spp.) [53].

### Evaluation of Pawlowsky gland infection status on louse-bite transmission efficiency

Groups of roughly 100 body lice were infected as described above, and, 6 days later, lice were screened for mCherry expression using fluorescent microscopy. Groups of 5 to 6 lice, diagnosed with a Pawlowsky gland or midgut only infection via detection of mCherry, were isolated for transmission assays. Transmission to the blood reservoir was determined as described previously for an initial extended (20 h) and subsequent short (3 h) feeding period. At the end of the 2 transmission assays, lice were frozen at −80°C for bacterial enumeration. Following surface sterilization, the head of each louse was separated from the body using microscissors, and both structures were mechanically disrupted and plated separately using BHI soft agar overlays as described above.

### Immunohistochemistry of infected lice

Groups of sterile or infected lice with detectable mCherry signal in the head were transferred to a drop of PBS, their cuticle was punctured 3 times with an insect pin, and lice were transferred to 10% neutral buffered formalin and fixed for at least 24 h. Fixed lice were processed using the VIP-6 Tissue Tek processor (Sakura Finetek USA) and then embedded in paraffin. Blocks were sectioned at 5 μm and every third section was stained with hematoxylin and eosin to identify the desired regions for IHC. Labeling of *Y*. *pestis* was performed with a polyclonal α-*Y*. *pestis* antibody [54] (rabbit; 1:4,000) and a secondary α-rabbit-HRP conjugate (Vector Laboratories ImmPRESS-VR Horse anti-rabbit IgG polymer). IHC was performed using the Discovery purple kit and the Roche/Ventana Discovery ULTRA staining platform according to manufacturer’s instructions (Roche Tissue Diagnostics). Slides were counterstained with hematoxylin and *Y*. *pestis* appears pink or purple following staining with the Discovery chromogen.

## Supporting information

S1 Fig *Y*. *pestis* fecal shedding for direct- and delayed-transfer infected louse groups. *Y*. *pestis* KIM6+ CFU recovered from louse feces following the (A) long, 20 h or (B) short, 3-h feeding period. Each dot represents data from 1 of the 3 experiments from direct- or delayed-transfer groups of infected body lice described in Fig 1. Horizontal bars represent the mean, and all values are normalized to the number of lice feeding during that time period. *n* = 40 lice per group. Summary data for this figure can be found in S4 Data.
(TIF)

S2 Fig *Yersinia pestis* and *Yersinia pseudotuberculosis*, but not *Escherichia coli*, can colonize the Pawlowsky glands and be transmitted by body lice. (**A**) The infection rate and (**B**) bacterial burden for groups of body lice fed on blood containing 4.2–8.5 × 10^8^ CFU/ml of (1) *Y*. *pestis*: KIM6+, KIM6+*ymtH188N*, KIM6+*ΔhmsH*, CO92 (pCD1); (2) *E*. *coli* DH5α; or (3) *Y*. *pseudotuberculosis* IP32953. Horizontal bars represent the median and dashed line represents the limit of detection. Lice were screened by fluorescence microscopy on days 3, 5, and 7 postinfection to determine the percentage of lice that had bacteria in (**C**) the midgut (MG) or (**D**) the Pawlowsky glands (PG). CFUs recovered from the (**E**) feces or (**F**) from the blood reservoir at the end of a 20-h feeding period. Data are pooled from 1 (*Yersinia* spp. infections) or 3 (*E*. *coli*) independent experiments with *n* = 44–50 body lice. For graphs C–F, data from the 3 *E*. *coli* experiments are plotted individually. Infection rate and bacterial burden were determined from 6–10 individual mixed-sex lice per experiment. CFU data from feces and blood are normalized to the number of lice in the capsule for that day. Summary data for this figure can be found in S5 Data.
(TIF)

S3 Fig *Y*. *pseudotuberculosis* causes higher rates of louse mortality and frequently induces blood to leak into the hemocoel shortly before death. (**A**) Survival curve of body lice infected with different gram-negative bacteria as described in S2 Fig. *p < 0.01 by Log-rank test compared to *Y*. *pestis* KIM6+; *n* = 44–50. (**B**) Examples of body louse coloration of a healthy uninfected control (left) and a moribund louse infected with *Y*. *pseudotuberculosis* IP32953 (right). The red coloration indicates damage to the midgut epithelium and subsequent leakage of blood from the digestive tract into the hemocoel. Summary data for this figure can be found in S6 Data.
(TIF)

S1 Table Strains and plasmids used in this study. (DOCX)

S1 Data Numerical data used for Fig 1. (XLSX)

S2 Data Numerical data used for Fig 2. (XLSX)

S3 Data Numerical data used for Fig 3. (XLSX)

S4 Data Numerical data used for S1 Fig. (XLSX)

S5 Data Numerical data used for S2 Fig. (XLSX)

S6 Data Numerical data used for S3 Fig. (XLSX)

## Abbreviations

BHI: brain heart infusion
IHC: immunohistochemistry
PG: Pawlowsky gland
