# Supplementary figures and images for: Yersinia pestis can infect the Pawlowsky glands of human body lice and be transmitted by louse bite
Source: PLoS Biol. 2024 May 21;22(5):e3002625. doi: 10.1371/journal.pbio.3002625 (PMC11108126; doi:10.1371/journal.pbio.3002625)

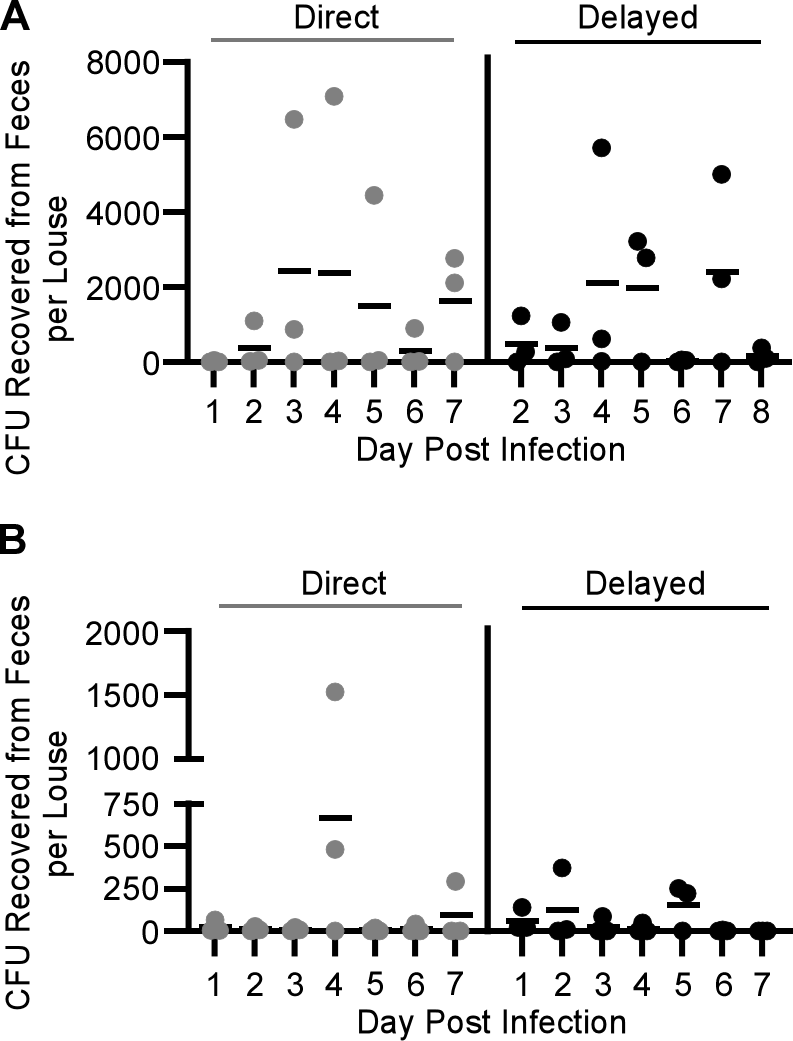

Supplement: S1 Fig — Y. pestis KIM6+ CFU recovered from louse feces following the (A) long, 20 h or (B) short, 3-h feeding period. Each dot represents data from 1 of the 3 experiments from direct- or delayed-transfer groups of infected body lice described in Fig 1. Horizontal bars represent the mean, and all values are normalized to the number of lice feeding during that time period. n = 40 lice per group. Summary data for this figure can be found in S4 Data. (TIF) [file pbio.3002625.s001.tif]

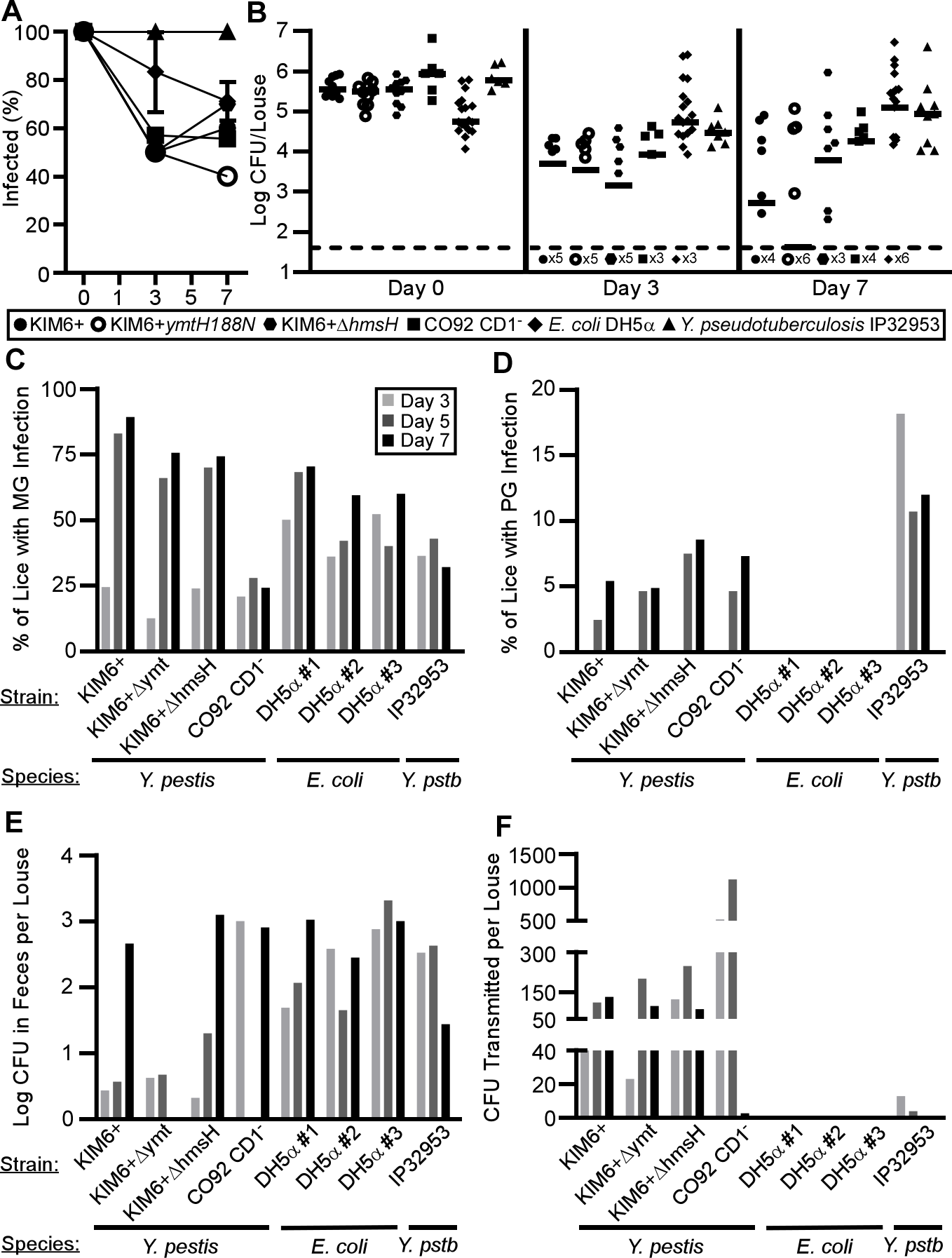

Supplement: S2 Fig — (A) The infection rate and (B) bacterial burden for groups of body lice fed on blood containing 4.2–8.5 × 10^8^ CFU/ml of (1) Y. pestis: KIM6+, KIM6+ymtH188N, KIM6+ΔhmsH, CO92 (pCD1); (2) E. coli DH5α; or (3) Y. pseudotuberculosis IP32953. Horizontal bars represent the median and dashed line represents the limit of detection. Lice were screened by fluorescence microscopy on days 3, 5, and 7 postinfection to determine the percentage of lice that had bacteria in (C) the midgut (MG) or (D) the Pawlowsky glands (PG). CFUs recovered from the (E) feces or (F) from the blood reservoir at the end of a 20-h feeding period. Data are pooled from 1 (Yersinia spp. infections) or 3 (E. coli) independent experiments with n = 44–50 body lice. For graphs C–F, data from the 3 E. coli experiments are plotted individually. Infection rate and bacterial burden were determined from 6–10 individual mixed-sex lice per experiment. CFU data from feces and blood are normalized to the number of lice in the capsule for that day. Summary data for this figure can be found in S5 Data. (TIF) [file pbio.3002625.s002.tif]

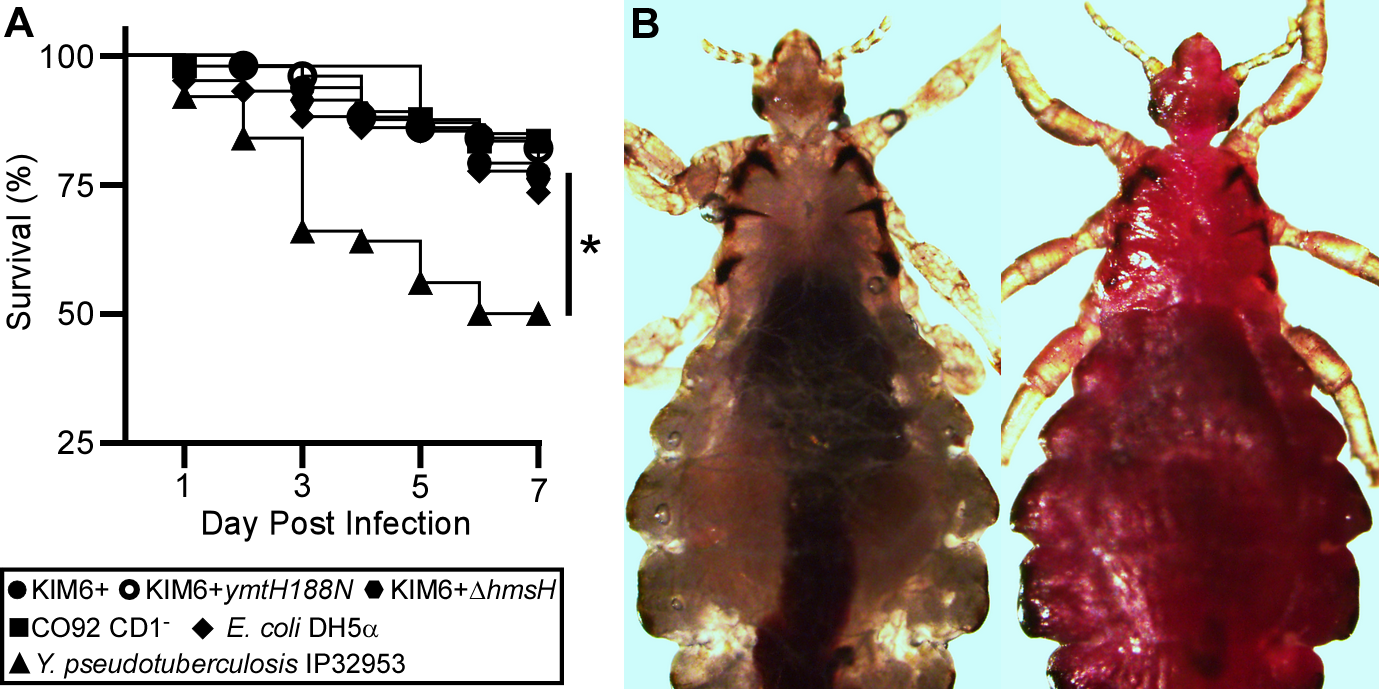

Supplement: S3 Fig — (A) Survival curve of body lice infected with different gram-negative bacteria as described in S2 Fig. *p < 0.01 by Log-rank test compared to Y. pestis KIM6+; n = 44–50. (B) Examples of body louse coloration of a healthy uninfected control (left) and a moribund louse infected with Y. pseudotuberculosis IP32953 (right). The red coloration indicates damage to the midgut epithelium and subsequent leakage of blood from the digestive tract into the hemocoel. Summary data for this figure can be found in S6 Data. (TIF) [file pbio.3002625.s003.tif]
