# Supplementary material for: Yersinia pestis can infect the Pawlowsky glands of human body lice and be transmitted by louse bite
Source: PLoS Biol. 2024 May 21;22(5):e3002625. doi: 10.1371/journal.pbio.3002625 (PMC11108126; doi:10.1371/journal.pbio.3002625)
Supplement: S1 Table — (DOCX) [file pbio.3002625.s004.docx]

**S1 Table. Bacterial Strains and Plasmids**

| **Strain/Plasmid** | **Key Properties** | **Reference/Source** |
| --- | --- | --- |
| ***Y. pestis* strains** |  |  |
| KIM6+ | pCD1^-^, pMT1+, pPCP1+, Pgm+, Biovar Medievalis | (1, 2) |
| KIM6+*ymtH188N* | KIM6+ modified to express a non-functional Ymt with a point mutation in one of the two HKD catalytic domains. | (3) |
| KIM6+Δ*hmsH* | Isogenic deletion of the *hmsH* open reading frame | (4) |
| CO92 CD1^-^ | pCD1^-^, pMT1+, pPCP1+, Pgm+, Biovar Orientalis | (5) |
| ***Y. pseudotuberculosis* strains** |  |  |
| IP32953 | O1:b serotype, strain most closely related to *Y. pestis* (44) | E. Carniel, Institut Pasteur, Paris, France (6) |
| ***E. coli* strains** |  |  |
| DH5α | Cloning strain | (7) |
| **Plasmids** |  |  |
| pmCherry | Ap^r^, constitutively expresses the mCherry fluorescent protein | Clontech/Takara Bio (Mountain View, CA) |
| pCD1 virulence plasmid, encodes type 3 secretion system  pMT1 plasmid, encodes the phospholipase D Yersinia murine toxin (Ymt) and capsule antigen (F1)  pPCP1 plasmid, encodes plasminogen activator/protease (Pla), the bacteriocin pesticin (Pst), and pesticin immunity protein (Pim)  Pgm pigmentation locus and pathogenicity island, encodes the hemin storage locus (*hmsHFRS* operon) and iron acquisition genes  Ap^r^ ampicillin resistance | | |

**References**

1. R. D. Perry, J. D. Fetherston, *Yersinia pestis*--etiologic agent of plague. *Clin. Microbiol. Rev.* **10**, 35-66 (1997).

2. D. J. Sikkema, R. R. Brubaker, Resistance to pesticin, storage of iron, and invasion of HeLa cells by Yersiniae. *Infect. Immun.* **55**, 572-578 (1987).

3. A. E. Rudolph *et al.*, Expression, characterization, and mutagenesis of the *Yersinia pestis* murine toxin, a phospholipase D superfamily member. *J. Biol. Chem.* **274**, 11824-11831 (1999).

4. C. L. Mitchell *et al.*, A role for early-phase transmission in the enzootic maintenance of plague. *PLoS Pathog.* **18**, e1010996 (2022).

5. J. Parkhill *et al.*, Genome sequence of *Yersinia pestis*, the causative agent of plague. *Nature* **413**, 523-527 (2001).

6. P. S. Chain *et al.*, Insights into the evolution of *Yersinia pestis* through whole-genome comparison with *Yersinia pseudotuberculosis*. *Proc. Natl. Acad. Sci. U.S.A.* **101**, 13826-13831 (2004).

7. D. Hanahan, *DNA cloning: a practical approach*. D. M. Glover, Ed. (IRL Press, Oxford, United Kingdom, 1985).
